# Harming Ourselves and Defiling Others: What Determines a Moral Domain?

**DOI:** 10.1371/journal.pone.0074434

**Published:** 2013-09-11

**Authors:** Alek Chakroff, James Dungan, Liane Young

**Affiliations:** 1 Department of Psychology, Harvard University, Cambridge, Massachusetts, United States of America; 2 Department of Psychology, Boston College, Chestnut Hill, Massachusetts, United States of America; Ecole Normale Supérieure, France

## Abstract

Recent work has distinguished “harm” from “purity” violations, but how does an act get classified as belonging to a domain in the first place? We demonstrate the impact of not only the kind of *action* (e.g., harmful versus impure) but also its *target* (e.g., oneself versus another). Across two experiments, common signatures of harm and purity tracked with other-directed and self-directed actions, respectively. First, participants judged self-directed acts as primarily impure and other-directed acts as primarily harmful. Second, conservatism predicted harsher judgments of self-directed but not other-directed acts. Third, while participants delivered harsher judgments of intentional versus accidental acts, this effect was smaller for self-directed than other-directed acts. Finally, participants judged self-directed acts more harshly when focusing on the actor’s character versus the action itself; other-directed acts elicited the opposite pattern. These findings suggest that moral domains are defined not only by the kind of action but also by the target of the action.

## Introduction

People judge many different acts to be immoral, from terrorism to tax evasion, from murder to masturbation. Researchers have suggested that moral judgments hang together in “moral domains”, for example, “harm” (e.g., assault, humiliation), “fairness” (e.g., lying, cheating), and “purity” (e.g., eating taboo substances, committing incest), to name a few [1–4]. Here, we focus on folk intuitions about two domains: harm and purity.

The current work builds directly on existing evidence for organizing harm violations and purity violations into distinct moral domains. First, moral judgments across putatively distinct domains may derive from distinct affective responses [5–9] (but see 10). As prior work has shown, moral judgments of harm and purity violations are rooted in the emotional responses of anger and disgust, respectively [1,11–13]. We note that while some work shows violations of harm and fairness norms to elicit reactions of disgust as well [14–19], recent research suggests that disgust in response to harm violations may be more similar to anger, relative to disgust in response to purity violations [11,13,20,21]. Nevertheless, to account for semantic overlap of anger and disgust, here we assess the effects of our manipulations on disgust that are unrelated to the analogous effects on anger, and vice versa (for a similar method and discussion, see 11,13,20; see also General Discussion). Second, different moral values are endorsed to a different extent across different socio-cultural groups. In particular, social conservatives tend to judge purity violations more harshly, compared to liberals, whereas both groups judge harms similarly [3,7,22,23] (see also 24). Third, recent evidence reveals that moral judgments of harm and purity violations rely on distinct cognitive processes, as suggested by neural data [25]-[28] and patterns of behavioral responses (for reviews, see 4,29). Relative to judgments of harm violations, judgments of purity violations are influenced to a lesser extent by contextual information [21], information about the violator’s reasons for acting [30,31], and, of central importance for the current research, information about the violator’s intent [32,20].

### What makes an act a harm violation versus a purity violation?

While recent work has highlighted important differences between harm and purity judgments, an outstanding question is this: how do people perceive an action to be a harm violation or a purity violation in the first place? We suggest that “moral categorization” relies on a complex set of cues. We examine the features of an act that lead to its categorization as a harm violation or a purity violation.

Consider two moral violations featured in prior proposals [2,6]: “sticking a pin into the palm of a child” (*harm*), and “cooking and eating human flesh” (*purity*). While both actions may be considered immoral, they differ along a number of dimensions. An obvious difference is that one act is physically *harmful*, while the other act is *impure* or *defiling*. A closer look at these examples though reveals that these violations also differ, implicitly, in terms of the *target* of the act–in other words, not simply what is being done but to whom–the child versus one’s own self (cf. [33]). The “harm violation” is dyadic or other-directed, involving an agent (violator) acting on a target (victim) [33,34]. By contrast, the “purity violation” is not obviously dyadic. Assuming that the human flesh was privately obtained from an individual who was already deceased, the cannibalistic act appears private and, importantly, immoral even under these restricted conditions; one’s own self represents both violator and “victim” of the act. Even when purity violations involve more than one person, i.e., incest between two consenting adults, it is not necessarily the case that one of them is a victim. Often the agents themselves are the only ones who are directly affected by their own impure or defiling actions, so there are no other victims. Notably, and, foreshadowing our hypotheses, researchers have specifically designed purity violations to be non-dyadic in order to investigate moral intuitions that are not related to perceived harm [6,20,21].

The present hypothesis is that the target or victim of an act plays a critical additional role in determining how moral violations are processed. One specific prediction is that *other-directed* violations (both harmful and impure acts) are processed as relatively more *harmful*, whereas *self-directed* violations (both harmful and impure acts) are processed as relatively more *impure*.

These predictions can be understood within a biological or cultural evolutionary perspective on moral domains: intuitions across moral domains may have evolved to address distinct adaptive challenges. For example, harm norms may serve to regulate interpersonal behavior [2,34], whereas purity norms may serve to protect oneself from exposure to pathogens (e.g., via food or sex) [1,15,21,35–37]. Thus, harm norms might apply to dyadic interactions or other-directed actions (i.e., a violator acts on a victim) as opposed to self-directed actions [33]. Conversely, purity norms might apply to a broader set of behaviors that are potentially harmful to the self [15,24,36,38–40].

The proposed function of purity norms also hints at an explanation for the idiosyncrasies of purity judgments found in prior research, as outlined above. If a person is infected with a contagious disease, our judgment of that person as dangerous need not depend on the person’s innocent intent or other contextual factors (cf. [20,32]). It is enough for us to know that the person is “tainted” and ought to be avoided [1] - note though that intuitions supporting pathogen avoidance are not always moralized (see 37 for discussion). By contrast, intent information may be critical for assigning moral blame to a person for having caused harm to others [41–43], as well as for predicting a person’s future harmful behaviors [44].

### The Present Research

We examined the impact of the target of an action (i.e., oneself versus another person) in determining moral domain membership. We relied on key behavioral “signatures” used in prior work and described above for distinguishing between domains.

#### Perceived Harmfulness and Impurity

First, “harm violations” are judged primarily as harmful, whereas “purity violations” are judged primarily as impure [13]. While we expected to find this same pattern in our data, we additionally predicted that *self-directed* acts (both harmful and impure) would be judged primarily as impure, while *other-directed* acts (both harmful and impure) would be judged primarily as harmful.

#### Social Conservatism

Second, social conservatism has been shown to influence moral judgment, such that conservatives versus liberals tend to judge “purity violations” as morally worse [3]. Judgments of “harm violations” differ less across the political spectrum; liberals and conservatives alike appear to agree that harms are morally wrong. We predicted that social conservatism would correlate with moral judgment to a greater extent for *self-directed* acts versus *other-directed* acts.

#### The Role of Intent

Third, intentional violations are typically judged as morally worse than accidental violations; however, the magnitude of this difference differs across domains. Specifically, intent plays a larger role in judgments of “harm violations” relative to judgments of “purity violations” [20,32]. We predicted that in addition intent also plays a larger role in judgments of *other-directed* versus *self-directed* acts.

#### Moral Focus on Action versus Character

Fourth, prior work has found that people judge some *acts* to be morally wrong, whereas they judge other acts as more informative of the actor’s poor moral *character* [45,46] (see also 47). We hypothesized that, compared to judgments of “harm violations” and *other-directed acts*, judgments of “purity violations” and *self-directed acts* reflect more poorly on the agent’s moral character, and less on the moral status of the act itself. We thus explored interactions among the following variables: action type (harmful versus impure), target (other-directed versus self-directed), and moral focus (whether participants made moral judgments focusing on the action versus character).

## Experiment 1: Self-directed Acts, Purity, and Intent

Experiment 1 tested our four key predictions within a single paradigm. Participants made moral judgments, as well as judgments of perceived harmfulness and impurity, of acts that varied along the following dimensions: (1) action: whether the act was defined a priori as harmful or impure (e.g., physical harm vs. exposure to bodily fluids), (2) target: whether the act was other-directed or self-directed, (3) intent: whether the act was intentional or accidental, and (4) moral focus: whether participants made judgments of the action or character.

### Method

#### Participants

We recruited 410 participants (254 male; *M*_*age*_ = 29.2, *SD*_*age*_ = 9.3) using Amazon Mechanical Turk (www.mturk.com). Participants were English speakers from the United States and paid sixteen cents for their time. 79 participants (19%) were excluded from analysis for failing an attention check (i.e., to recall the name of the protagonist in the scenarios). Both Experiments 1 and 2 were approved by the Boston College Internal Review Board. In both experiments, participants completed an IRB-approved consenting process, viewing an online consent document and indicating their consent via mouse click before proceeding to the tasks.

#### Procedure

In a 2 (action: harmful versus impure) x 2 (target: other-directed versus self-directed) x 2 (intent: intentional vs. accidental) between-subjects design, participants read four scenarios depicting a character committing an unethical act (e.g., Imagine that Steven intentionally punched someone in the ribs; see Text S2). For a single participant, all four scenarios depicted behavior from a single condition (e.g., harmful, other-directed, intentional).

For each scenario, participants made five judgments. Participants first provided a moral judgment either of the action (How morally wrong is this behavior?) or of the character (How immoral is Steven as a person?). Question type (i.e., action versus character) was randomized across scenarios within participants. The order of the next four measures was randomized across scenarios within participants: unnaturalness (How much does this violate the natural order of things - how unnatural is this?), disgust (How disgusted do you feel about this?), damage (How damaging is this?), and anger (How angry do you feel about this?). After rating all four scenarios, participants completed a brief demographics section, which included a single self-report measure of social conservatism. All ratings were made on 7-point Likert scales (see Text S2). Judgments were averaged across the four scenarios prior to analysis (Crohnbach’sα: Moral = .90; Unnatural = .86; Disgust = .87; Damage = .83; Anger = .88).

### Results and Discussion

We report results from four analyses of the data, each of which tested one of our four key predictions. We first report results from an analysis of judgments of perceived harmfulness and perceived impurity (*Prediction 1*). We then report the results of regression analyses predicting moral judgments from social conservatism (and experimental manipulations) (*Prediction 2*). Finally, we report results from an analysis of moral judgments, focusing on the interactions among the variables action, target, and intent (*Prediction 3*), and moral focus (*Prediction 4*).

#### Extracting Impurity and Harmfulness composites

Participants delivered judgments of unnaturalness and disgust [3,48], designed to measure perceived impurity, as well as damage and anger [3,13], designed to measure perceived harmfulness. Our a priori grouping of measures was confirmed by a principal components analysis conducted with Varimax rotation on participants’ judgments of unnaturalness, disgust, damage, and anger. The first two components accounted for 87.9% of the variance in the data. As shown in Table 1, rotated factor loadings show that the two highest-loading measures for the first component were unnaturalness and disgust, while the two highest-loading measures on the second component were damage and anger. Unnaturalness and disgust judgments (*r*(331) = .73, *p* < .001) were then averaged to produce a composite of perceived impurity, while anger and damage judgments (*r*(331) = .66, *p* < .001) were averaged to form a composite of perceived harmfulness. We report analyses of measures used to construct the composites in Text S1 and Figure S1.

**Table 1:** Harmfulness and Impurity Composite Factor Scores for measures in Experiment 1.

	*PC1*	*PC2*
Unnaturalness	**.75**	.52
Disgust	**.94**	.18
Damage	.25	**.95**
Anger	.69	**.58**

#### Prediction 1: Harmfulness and Impurity

“Harm violations” and “purity violations” are typically judged primarily as harmful and impure, respectively [13]. Here, we examined whether perceptions of harmfulness and impurity depend additionally on the target of the violation. We then conducted a 2 (action: harmful versus impure) x 2 (target: other-directed versus self-directed) x 2 (composite: harmfulness and impurity) mixed effects ANOVA.

#### Main Effects

Participants judged acts to be more impure (*M* = 4.6, *SE* = .12) than harmful (*M* = 4.2, *SE* = .12) on the whole (main effect of composite; *F*(1,327) = 25.79, *p* < .001, η_p_^2^ = .07). Participants also delivered “harsher” judgments (more impure and harmful) for impure acts (*M* = 4.7, *SE* = .11) versus harmful acts (*M* = 4.3, *SE* = .12) (main effect of action; *F*(1,327) = 6.9, *p* = .009, η_p_^2^ = .02). Finally, participants delivered “harsher” judgments (more impure and harmful) for other-directed acts (*M* = 4.8, *SE* = .11) versus self-directed acts (*M* = 4.2, *SE* = .11) (main effect of target; *F*(1,327) = 18.37, *p* < .001, η_p_^2^ = .05).

#### Action, Harmfulness, and Impurity

An interaction emerged between action and composite (*F*(1,327) = 106.05, *p* < .001, η_p_^2^ = .25), indicating that harmful acts were judged as more harmful (*M* = 4.4, *SE* = .12) than impure (*M* = 4.2, *SE* = .12) (*t*(149) = -3.78, *p* < .001), whereas impure acts were judged as more impure (*M* = 5.1, *SE* = .11) than harmful (*M* = 4.3, *SE* = .11) (*t*(180) = 10.47, *p* < .001). In addition, impure acts were judged as more impure relative to harmful acts (*M* = 4.2, *SE* = .12) (*t*(329) = 5.69, *p* < .001), while harmful and impure acts were judged as similarly harmful (*M* = 4.3, *SE* = .11) (*t*(329) = -.67, *p* = .5). This analysis served as a basic check of the action (harmful vs. impure) manipulation, while also supporting the validity of the impurity and harmfulness composites.

#### Target, Harmfulness, and Impurity

Given our hypothesis that the target of a moral violation determines how the violation is processed (i.e., as harmful or impure), we predicted links between self-directed violations and impurity judgments, and between other-directed violations and harmfulness judgments. Consistent with this prediction, we found a target x composite interaction (*F*(1,327) = 12.35, *p* = .001, η_p_^2^ = .04). Participants judged other-directed acts to be similarly impure (*M* = 4.9, *SE* = .12) and harmful (*M* = 4.8, *SE* = .11) (*t*(168) = 1.86, p = .06), but, critically, participants judged self-directed acts to be more impure (*M* = 4.4, *SE* = .13) than harmful (*M* = 3.9, *SE* = .12) (*t*(161) = 5.10, *p* < .001).

Because the harmfulness and impurity composites are highly related (*r*(331) = .76, *p* < .001), it is also useful to examine the unique effects of our manipulations on each composite, controlling for the effects on the other composite (see 11,13,20).

**Figure 1:**
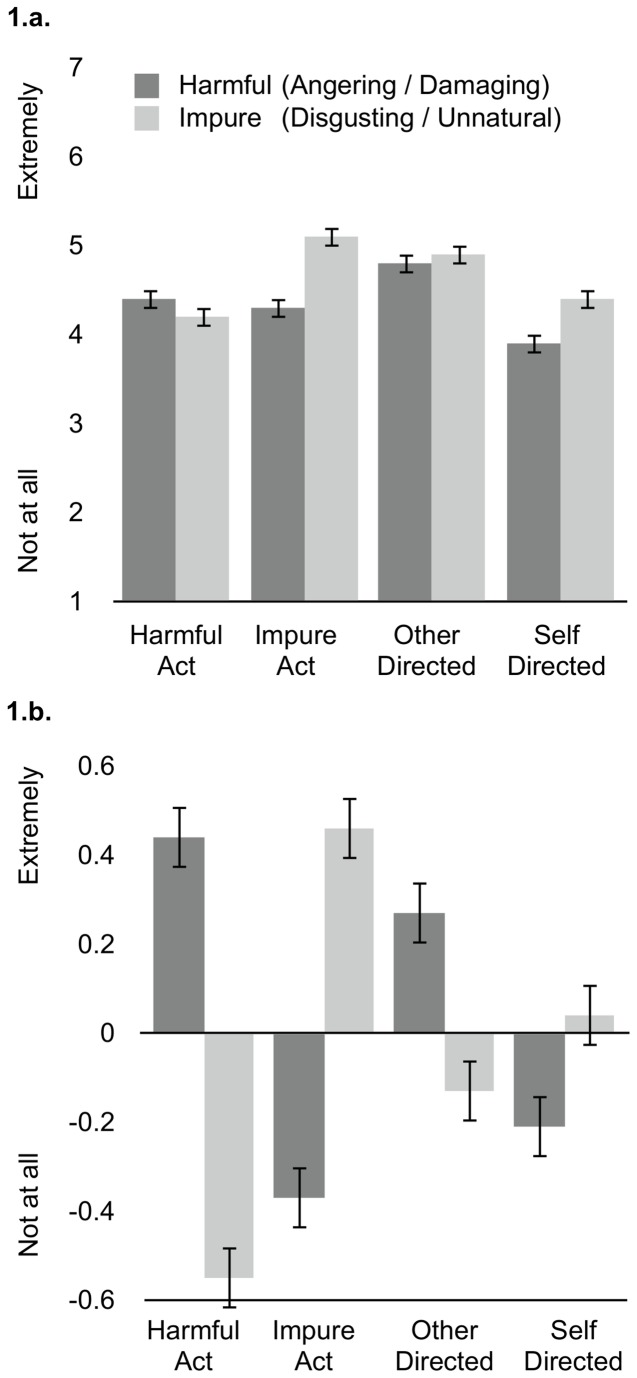
Perceived Harmfulness and Impurity. Composite judgments of harmfulness (dark bars) and impurity (light bars), in raw form (1.a.) as well as standardized, controlling for effects on the opposing composite (1.b.). Error bars represent ± 1 SE.

We thus conducted the same ANOVA presented above on composites that measured unique variance in harmfulness and impurity. We computed these composites as the standardized residual values in impurity, predicted from harmfulness, and vice versa. Effects of action and target on each composite are shown in Figure 1. As above, an interaction emerged between action and composite (*F*(1,327) = 104.49, *p* < .001, η_p_^2^ = .24), indicating that harmful acts were judged as more harmful (*M* = .44, *SE* = .07) than impure (*M* = -.55, *SE* = .07) (*t*(149) = -8.06, *p* < .001), and also more harmful compared to impure acts (*M* = -.37, *SE* = .07) (*t*(329) = -8.0, *p* < .001). By contrast, impure acts were rated as more impure (*M* = .46, *SE* = .06) than harmful (*M* = -.37, *SE* = .07) (*t*(180) = 6.29, *p* < .001), and also as more impure compared to harmful acts (*M* = -.55, *SE* = .07) (*t*(329) = 10.47, *p* < .001).

In addition, as above, an interaction emerged between target and composite (*F*(1,327) = 13.17, *p* < .001, η_p_^2^ = .04). Participants judged other-directed acts to be more harmful (*M* = .27, *SE* = .07) than impure (*M* = -.13, *SE* = .07) (*t*(168) = -2.73, p = .007), and also more harmful compared to self-directed acts (*M* = -.21, *SE* = .07) (*t*(329) = 4.56. *p* < .001). By contrast, participants judged self-directed acts to be more impure (*M* = .04, *SE* = .07) than harmful (*M* = -.21, *SE* = .07) (*t*(161) = 2.0, *p* = .047), and also trended toward judging self-directed acts as more impure compared to other-directed acts (*M* = -.13, *SE* = .07) (*t*(329) = -1.52. *p* = .13).

Notably, comparing conditions that share “features” (i.e., action, target) of both harm and purity violations reveals a key role for target in determining domain membership. Specifically, self-directed harmful acts were judged as more impure (*M* = .32, *SE* = .09) than harmful (*M* = -.07, *SE* = .09) (*t*(92) = 2.59, *p* = .01); self-directed harmful acts were also judged as more impure than other-directed impure acts (*M* = -.53, *SE* = .11) (*t*(165) = 5.99, *p* < .001). By contrast, other-directed impure acts were judged as more harmful (*M* = .26, *SE* = .10) than impure (*M* = -.53, *SE* = .11) (*t*(73) = -3.75, *p* < .001); other-directed impure acts were also judged as more harmful than self-directed harmful acts (*M* = -.07, *SE* = .09) (*t*(165) = -2.50, *p* = .01).

#### Action and Target

There were no significant interactions between action and target (*F*(1,327) = .004, *p* = .95, η_p_^2^ < .001), or between action, target and composite (*F*(1,327) = 1.83, *p* = .18, η_p_^2^ < .01), suggesting that action type (harmful versus impure) and target (other-directed versus self-directed) play independent roles in determining moral domain.

#### Prediction *2*: Social Conservatism

While social conservatives and liberals alike agree that harm violations are immoral, they do not necessarily agree as to whether purity violations (e.g., eating one’s dead dog) are immoral. Recent research indicates that social conservatism predicts harsher judgments of purity violations but not harm violations [3,7,49]. We therefore examined whether social conservatism would also interact with the target of moral violations. For example, would social conservatism predict harsher moral judgments of self-directed acts in particular? We conducted a linear regression predicting moral severity from self-reported social conservatism, action (harmful versus impure), target (other-directed versus self-directed), intent (intentional versus accidental), and judgment type (action versus character), while also probing interaction effects between conservatism and the four manipulations.

#### Action, Target, and Social Conservatism

The predictors explained 34% of the variance in moral judgment (*F*(9,321) = 18.20, *p* < .001). As shown in Table 2, we found significant effects of target and intent, such that other-directed acts were judged as worse than self-directed acts, and intentional acts were judged as worse than accidental acts. There were no unique predictive effects for action or moral focus. Greater conservatism predicted harsher moral judgments overall. We found no interaction between conservatism and action, indicating that conservatism predicted moral judgment to a similar extent for harmful and impure acts. However, in line with our novel prediction, we found a significant interaction between conservatism and target, indicating that greater conservatism predicted harsher moral judgments, to a greater extent for self-directed acts relative to other-directed acts. Notably, although other-directed acts were rated as worse than self-directed acts in general, judgments were still well below the maximum rating (M = 4.7 out of 7). Response variance was also similar for moral judgments of self-directed and other-directed acts (SE = .13 for both). Thus, the low predictive power of conservatism for moral judgments of other-directed acts does not appear to be due to a ceiling effect or reduced variance in responses. Finally, we also found a significant interaction between conservatism and intent, indicating that greater conservatism predicted harsher moral judgments to a greater extent for accidental acts relative to intentional acts. In other words, intent played a smaller role in the moral judgments of social conservatives versus liberals.

**Table 2:** Predictors of moral judgment in Experiments 1 & 2.

	*B*	*SE B*	*Beta*	*t*	
*Experiment 1*					
Action	0.17	0.39	0.04	0.44	
Target	-2.18	0.39	-0.57	-5.57	**
Intent	2.29	0.40	0.60	5.76	*
Moral Focus	0.02	0.40	0.01	0.05	
Conservatism	0.25	0.12	0.23	2.13	‡
Conservatism x Action	-0.13	0.10	-0.14	-1.25	
Conservatism x Target	0.33	0.10	0.36	3.13	*
Conservatism x Intent	-0.22	0.10	-0.23	-2.08	‡
Conservatism x Moral Focus	0.12	0.10	0.12	1.12	
*Experiment 2*					
Action	-9.59	6.11	-0.20	-1.57	
Target	-48.35	6.00	-1.00	-8.05	**
Conservatism	-1.69	1.75	-0.10	-0.97	
Conservatism x Action	1.13	1.97	0.08	0.57	
Conservatism x Target	5.38	1.93	0.36	2.79	*

‡p < .05 *p < .01 **p < .001

We conducted a follow-up 2 (action) x 2 (target) x 2 (intent) x 2 (conservatism) ANOVA based on a median split of the data for conservatism (>3 versus <=3). Mirroring the regression results, this analysis revealed “harsher” judgments overall by participants high in conservatism (*M* = 4.44, *SE* = .13) versus low in conservatism (*M* = 3.69, *SE* = .13) (*F*(1,315) = 17.64, *p* < .001, η_p_^2^ = .05). Again, we found an interaction between target and conservatism (*F*(1,315) = 8.12, *p* = .005, η_p_^2^ = .03), such that self-directed acts were judged as morally worse by participants high in conservatism (*M* = 4.1, *SE* = .19) versus participants low in conservatism (*M* = 2.9, *SE* = .18) (*t*(160) = -3.68*, p* < .001), while other-directed acts were judged as similarly wrong by participants high in conservatism (*M* = 4.7, *SE* = .18) and low in conservatism (*M* = 4.5, *SE* = .17) (*t*(167) = .144*, p* = .88).

As in the regression results reported above, we found no action by conservatism interaction (*F*(1,315) = .01, *p* < .92, η_p_^2^ < .001). Finally, we found a marginal intent by conservatism interaction (*F*(1,315) = 3.35, *p* = .07, η_p_^2^ = .01): accidental acts were given “harsher” judgments by participants high in conservatism (*M* = 3.8, *SE* = .17) versus low in conservatism (*M* = 2.7, *SE* = .19) (*t*(162) = -4.14*, p* < .001), whereas intentional acts were judged similarly by participants high in conservatism (*M* = 5.1, *SE* = .20) versus low in conservatism (*M* = 4.6, *SE* = .16) (*t*(165) = -1.42*, p* = .16).

#### Prediction *3*: The Role of Intent in Moral Judgments

While intentional moral violations are generally judged as morally worse than accidental violations, previous research has shown a differential role of intent across moral domains [20,21]. We sought to examine whether the role of intent depends on the action and/or target of moral violations. We therefore conducted a 2 (action: harmful versus impure) x 2 (target: other-directed versus self-directed) x 2 (intent: intentional versus accidental) x 2 (moral focus: action versus character) ANOVA.

#### Main Effects

**Figure 2:**
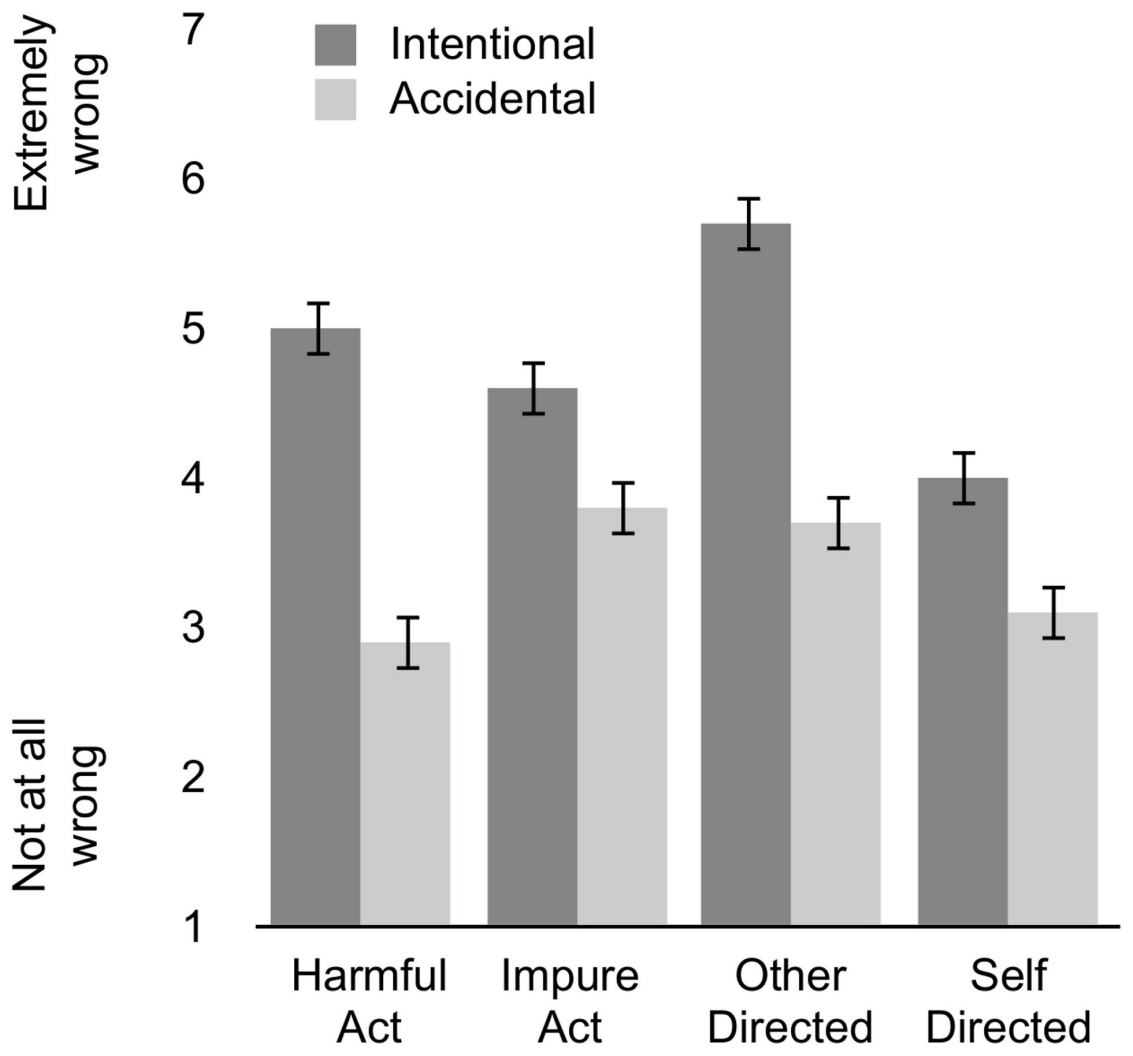
The Role of Intent. Moral severity for intentional acts (dark bars) and accidental acts (light bars). Ratings given for harmful and impure acts (collapsing across target), and for other-directed and self-directed acts (collapsing across action). Error bars represent ± 1 SE.

Effects of action, target, and intent on moral judgment are shown in Figure 2. As expected, participants judged intentional acts (*M* = 4.8, *SE* = .13) as morally worse than accidental acts (*M* = 3.4, *SE* = .13) (*F*(1,402) = 62.47, *p* < .001, η_p_^2^ = .17); participants also judged other-directed acts (*M* = 4.7, *SE* = .13) as morally worse than self-directed acts (*M* = 3.5, *SE* = .13) (*F*(1,402) = 39.55, *p* < .001, η_p_^2^ = .11). Finally, participants delivered harsher moral judgments when focusing on the action (*M* = 4.3, *SE* = .14) versus the character (*M* = 3.9, *SE* = .13) (*F*(1,315) = 4.19, *p* = .04, η_p_^2^ = .01).

#### Action, Target, and Intent

Replicating prior research [20,32], we found a larger effect of intent on moral judgments of harmful acts (*M*_*int*_ = 5.0, *SE* = .19; *M*_*acc*_ = 2.9, *SE* = .2) relative to impure acts (*M*_*int*_ = 4.6, *SE* = .18; *M*_*acc*_ = 3.8, *SE* = .18) (action x intent interaction; *F*(1,315) = 13.17, *p* < .001, η_p_^2^ = .04). Critically, and, in line with the current predictions, we also found a larger effect of intent on moral judgments of other-directed acts (*M*_*int*_ = 5.7, *SE* = .18; *M*_*acc*_ = 3.7, *SE* = .19) relative to self-directed acts (*M*_*int*_ = 4.0, *SE* = .19; *M*_*acc*_ = 3.1, *SE* = .19) (target x intent interaction; *F*(1,402) = 8.49, *p* = .004, η_p_^2^ = .03).

A follow-up ANOVA using data standardized separately within each condition (e.g., self-directed impure act) revealed the same pattern of effects, including the interaction between action and intent (*F*(1,323) = 12.36, *p* = .001, η_p_^2^ = .04), and the interaction between target and intent (*F*(1,323) = 10.12, *p* = .002, η_p_^2^ = .03).

#### Action and Target

As in the analysis of harmfulness and impurity composites, we did not find significant interactions between action and target (*F*(1,315) = 1.33, *p* = .25, η_p_^2^ < .01), or between action, target and intent (*F*(1,315) = .09, *p* = .77, η_p_^2^ < .001).

#### Prediction *4*: Action, Target, and Moral Focus

In the same 2 (action: harmful versus impure) x 2 (target: other-directed versus self-directed) x 2 (intent: intentional versus accidental) x 2 (moral focus: action versus character) ANOVA, we did not find support for our prediction that the focus of moral judgment (action versus character) would interact with target or action (all *p*’*s* > .05). The absence of an effect may be a consequence of the measures used; in particular, these measures have not been shown to reliably distinguish between moral judgment of an action on the one hand and judgment of moral character on the other hand. We address this issue below in Experiment 2.

#### Summary

First, we found that, on the whole, self-directed moral violations were judged as more impure, whereas other-directed violations were judged as more harmful. Second, we found that social conservatism predicted harsher moral judgments to a greater degree for self-directed versus other-directed acts. Finally, we found a larger role for intent in judging harmful versus impure acts and, importantly, in judging other-directed versus self-directed acts.

## Experiment 2: Self-directed Acts and Moral Character

In Experiment 1, we did not find support for the hypotheses that self-directed or impure acts indicate poor moral character, while other-directed or harmful acts reflect poorly on the moral status of an action. The absence of this predicted difference might be due to the measures we used, as these measures have not been previously shown to distinguish between judgments of an action and judgments of character. In Experiment 2, we adapted measures used effectively in prior work to distinguish between moral assessments of action versus character [46].

### Method

#### Participants

We tested 166 participants (116 male; *M*_*age*_ = 27.2, *SD*_*age*_ = 9.1) using Amazon Mechanical Turk (www.mturk.com). Participants were English speakers from the United States and paid twenty-six cents for their time. 10 participants (6%) were excluded from analysis for failing an attention check. Notably, the attention check in Exp 2 was disguised as a question in the demographics section asking about the participant’s favorite hobbies. If participants read the instructions, they would know to indicate “none of the above”. The smaller exclusion percentage in Exp 2 (6%) versus Exp 1 (19%) may be due to the particular attention checks used or different payment amounts.

#### Procedure

We tested our hypothesis using a 2 (action: harmful versus impure) x 2 (target: other-directed versus self-directed) x 2 (moral focus: action versus character) mixed-effects design. Participants read two scenarios depicting a character committing an unethical act (e.g., John once cut someone with a knife when he was upset; see Text S2). Unlike Experiment 1, no intent information was explicitly provided. As in Experiment 1, participants judged scenarios from a single condition (e.g., harmful, other-directed).

For each scenario, participants made three judgments of the moral status of the act (e.g., Were these actions immoral?) and three judgments of the moral status of the character (e.g., Is John “sick and twisted”?), using 100-point slider scales (cf. [46]; see Text S2). Judgments of act and character were made in separate blocks; block order was randomized across scenario. Judgments were averaged across the three items and two scenarios to create composite moral judgments focusing on either the action (α = .93) or character (α = .85). As in Experiment 1, after rating all four scenarios, participants completed a brief demographics section, which included a self-report measure of social conservatism on a 7-point Likert scale.

### Results and Discussion

We conducted a 2 (action: harmful versus impure) x 2 (target: other-directed versus self-directed) x 2 (moral focus: action versus character) mixed-effects ANOVA.

#### Main Effects

**Figure 3:**
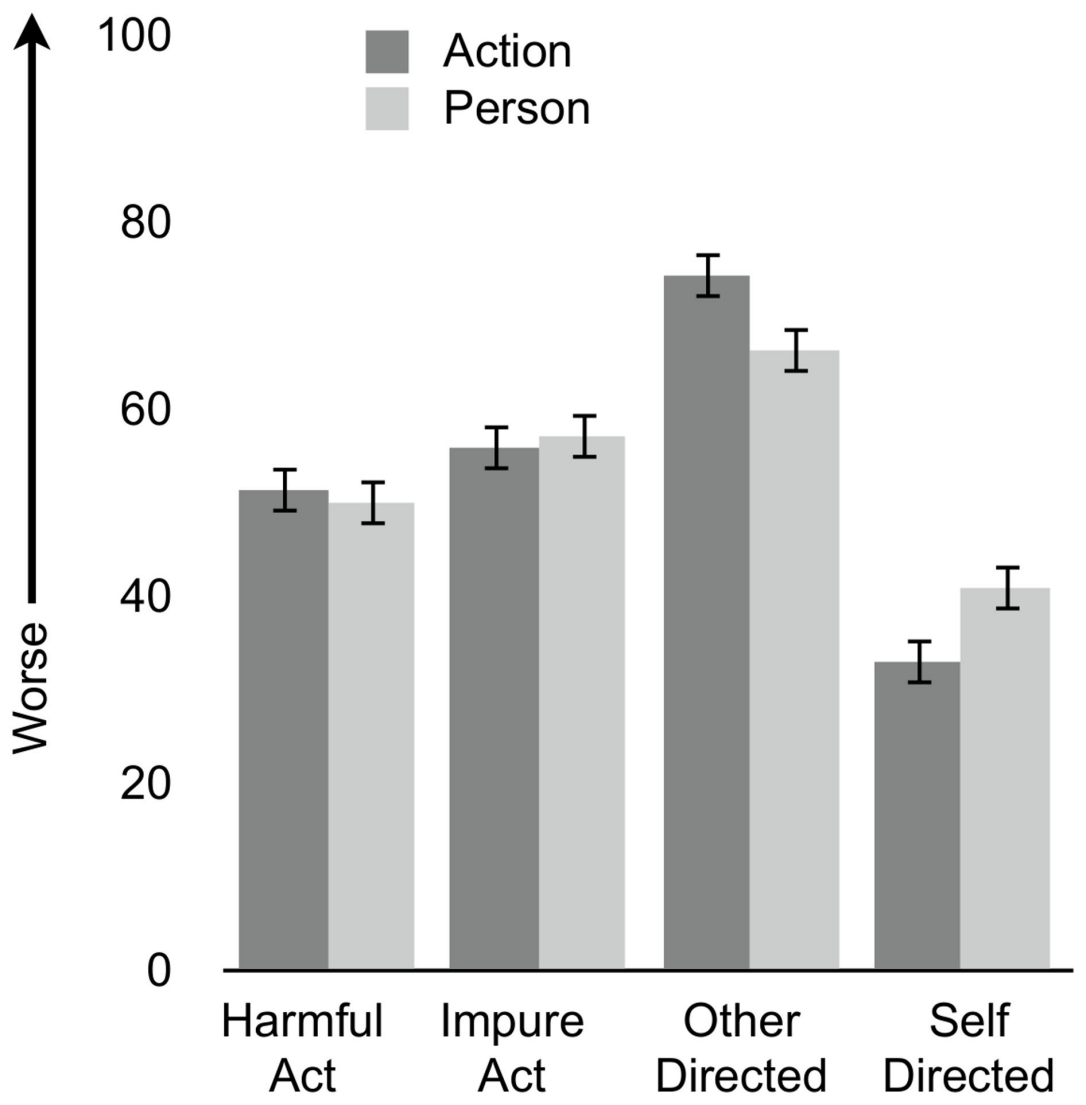
Moral Judgments of Action and Character. Moral judgments delivered when focusing on action (dark bars) and character (light bars). Ratings given for harmful and impure acts (collapsing across target), and for other-directed and self-directed acts (collapsing across action). Error bars represent ± 1 SE.

Effects of action, target, and moral focus on moral judgment are shown in Figure 3. As in Experiment 1, participants delivered harsher judgments for impure acts (*M* = 56.5, *SE* = 2) versus harmful acts (*M* = 50.7, *SE* = 1.9) (main effect of action; *F*(1,152) = 4.46, *p* = .036, η_p_^2^ = .03). Also, as in Experiment 1, participants delivered harsher judgments for other-directed acts (*M* = 70.2, *SE* = 1.9) versus self-directed acts (*M* = 36.9, *SE* = 2) (main effect of target; *F*(1,152) = 146.5, *p* < .001, η_p_^2^ = .49). Unlike Experiment 1, participants delivered similarly harsh judgments when focusing on the character (*M* = 53.6, *SE* = 1.5) and action (*M* = 53.6, *SE* = 1.8).

#### Action, Target, and Moral Focus

As in Experiment 1, there was no significant interaction between action and moral focus (*F*(1,152) = .47, *p* = .49, η_p_^2^ = .003). Critically, an interaction emerged between target and moral focus (*F*(1,152) = 17.81, *p* < .001, η_p_^2^ = .11), indicating that self-directed acts elicited harsher judgments of character (*M* = 40.9, *SE* = 2.2) than action (*M* = 33.0, *SE* = 2.6) (*t*(75) = -2.6, *p* = .01), whereas other-directed acts elicited harsher judgments of action (*M* = 74.3, *SE* = 2.5) than character (*M* = 66.3, *SE* = 2.1) (*t*(79) = 3.6, *p* < .001). We also found a marginal interaction between action and target (*F*(1,152) = 3.47, *p* = .06, η_p_^2^ = .02), such that impure acts were judged as morally worse than harmful acts, more for self-directed acts compared to other-directed acts. Finally, there was no interaction between action, target and moral focus (*F*(1,152) = .83, *p* = .36, η_p_^2^ < .01).

#### Action, Target, and Social Conservatism

As in Experiment 1, we conducted a linear regression predicting moral judgments (averaging across action and character judgments) based off of the action and target manipulations, self-reported social conservatism, and interaction effects between conservatism and experimental manipulations. The predictors, shown in Table 2, explained 53% of the variance in moral judgment (*F*(5,150) = 34.06, *p* < .001). As in Experiment 1, target predicted moral judgments (i.e., participants delivered harsher judgments of other-directed acts versus self-directed acts); action (i.e., harmful versus impure) was not a significant predictor of moral judgment. Conservatism was not a unique predictor of moral severity, and there was no interaction between action and conservatism. Critically, as in Experiment 1, there was an interaction between target and conservatism, such that conservatism was a better predictor of moral judgment for self-directed acts versus other-directed acts.

#### Summary

Using previously validated measures, we found support for the hypothesis that participants judge *self-directed acts* more harshly when focusing on the character of the actor versus the moral status of the action. By contrast, participants judged *other-directed acts* more harshly when focusing on the action versus character. There was no significant interaction between moral focus (character vs. action) and the type of action performed (impure vs. harmful). Finally, as in Experiment 1, social conservatism predicted harsher moral judgments to a greater degree for self-directed acts versus other-directed acts.

## General Discussion

Across two experiments, we found support for the account that the target of a moral violation (e.g., oneself versus other person) influences whether that violation is processed as a harm violation or a purity violation. This feature of target influenced moral judgment in the expected direction, for separate behavioral signatures established in prior work to reflect key differences in the processing of harm versus purity violations. Before we discuss implications of the present work, we offer a brief summary of the key findings.

First, in Experiment 1, participants judged self-directed violations as relatively more impure than harmful. By contrast, participants judged other-directed violations as relatively more harmful than impure. Second, across both experiments, self-reported social conservatism uniquely predicted harsh moral judgments of self-directed acts, but not other-directed acts. Third, in Experiment 1, we found a larger role for intent in moral judgments of other-directed versus self-directed acts: intentionally acting on another person was judged morally worse than accidentally acting on the person, but this difference was smaller for acts directed toward the self. We also found a smaller role of intent for judgments of harmful versus impure acts, mirroring prior work [20,32]. Finally, using validated dependent measures in Experiment 2, participants provided harsher moral judgments of character versus action for self-directed acts; the opposite pattern emerged for other-directed acts.

The present findings are consistent with the proposed adaptive functions of harm and purity norms [4,29,40]. If harm norms regulate interpersonal behavior, they may apply uniquely to behavior involving two or more individuals [33,34]. Indeed, any other-directed negative behavior may be construed as harmful, even if actual physical harm is absent [41,49–51]. Conversely, if purity norms ultimately serve to protect the self from pathogens (and consequent harm) [1,15,21,24,35–37], they may relate to any behavior that can be seen as harmful to oneself, even if the behavior does not contain typical elicitors of “bodily” disgust (for discussion see 21,22,39). This feature of purity norms appears to obtain even when people judge *other people*’s self-directed actions, perhaps because people simulate those self-directed actions [52].

Our account faces two specific challenges from competing models of moral emotions (e.g., [16]). The first challenge is for our claim that disgust is specifically associated with purity violations or self-directed acts. As noted in our introduction, prior work has shown that violations of harm and fairness may also elicit disgust [14–19]. If disgust is a general moral emotion, how can it be used to distinguish one moral domain from another? We suggest that moral violations of harm, fairness, and purity norms may all evoke multiple emotions (e.g., anger, disgust) to varying degrees. Recent research suggests that disgust is in fact a relevant emotion in the domain of harm, but not in comparison to anger [15,17,18], see 21 for discussion. Other work has found “moral disgust” to be even more morally relevant than anger [16]; however, follow-up work suggests that this difference may be due to the inclusion of the word “moral” as a qualifier for disgust but not anger [53]. Indeed, research that has measured both disgust and anger in response to harm and purity violations finds the pattern observed in the present data: both harm and purity violations elicit both anger and disgust, but harms are preferentially linked to anger, while purity violations are preferentially linked to disgust [11,13,20,53].

A second challenge comes from a recent social-functionalist account of moral emotions [16]. Moral anger is proposed to be an adaptive response to self-relevant immoral acts (e.g., I get angry if someone hits me), while moral disgust is the result of an appraisal of others who have immoral dispositions but who are not immediately threatening to us. We note that the “self / other” distinction in the social-functionalist account is distinct from the “self / other” distinction used in the present research. Moral interactions typically involve at least three “roles”-an agent (who commits the immoral act), a target (who is primarily affected), and a judge (who deems the act to be wrong) [54]. Different individuals may play different roles in a moral interaction, or the same individual may take on multiple roles. On the social-functionalist account, when the *target and judge* are the same person (e.g., A student steals *your* exam and copies it, and *you* judge the student for acting in this way), the immoral act is “self-relevant” and is associated with anger primarily; when the target and judge are different people (e.g., A student steals another student’s exam and copies it), the act is less self-relevant and is associated with moral disgust. On our account, when the *agent and target* are the same person (e.g., self-cutting), the act is self-directed and is associated with disgust and impurity; when the agent and target are different people (e.g., cutting someone else), the act is other-directed and associated with anger and harm. We note that these two accounts of moral emotions and the self / other distinction are not mutually exclusive, and future research should more directly explore the links between them.

The present research examined the unique role of target in determining domain membership, but we emphasize here our results do not show the target of a moral act is the primary determinant of moral domain membership. First, the type of action performed (harmful versus impure) played a critical role in determining domain membership in the current work. Collapsing across target, we found that harmful acts were still judged as more harmful than impure, and impure acts were judged as more impure than harmful. Second, we found a larger role of intent for harmful versus impure acts, as in prior work, again collapsing across target. Nevertheless, it is worth noting that comparing acts that shared features of both harm and purity violations (i.e., self-directed harm and other-directed impurity) revealed a key role of target in determining domain membership. For example, self-directed harmful acts were judged as more impure than harmful and also as more impure than other-directed impure acts. Meanwhile, other-directed impure acts were judged as more harmful than impure and also as more harmful than self-directed harmful acts.

One unpredicted finding was that action and target emerged as relatively independent predictors of our behavioral “signatures” of domain membership. In Experiment 1, we did not find any interactions that involved action and target, in either the analyses of harmfulness and impurity composites, or in the analyses of moral judgments. In Experiment 2, we found a marginal interaction between action and target, such that impure acts were judged as morally worse than harmful acts, more for self-directed acts compared to other-directed acts. However, we found no interaction between action, target, and moral focus. The general absence of interactions among variables suggests that people may process action and target independently when determining domain membership—whether an act is a harm violation or a purity violation.

The present work may shed light on why some acts appear to crosscut domain boundaries. Heinous harms may evoke disgust; indeed, the violator himself may appear to be contaminated by his actions, and his moral character may therefore seem contagious – e.g., nobody wants to wear Hitler’s sweater [1]. One account of this effect is that extreme harms are downright strange. When harmful acts are uncommon or bizarre, they may be judged as primarily reflective of poor moral character. One recent study features scenarios in which a man becomes angry with his girlfriend and either abuses her or abuses her cat. The latter act seems more out of the ordinary and thus carries more information about the perpetrator and his desires [45,46,55] (see also 16). Self-directed acts (both harmful and impure) may appear to be less typical, driving the specific effects associated with moral character judgments reported here. Notably, though, such effects ultimately derive from the manipulation of the *target* of the act, underscoring our suggestion that domain membership depends on more than simply the kind of action performed – not all immoral harmful acts are judged equivalently as harm violations.

Given potential overlap across moral domains, we suggest that domains do not necessarily represent discrete categories or natural moral “kinds”. Instead, moral judgment reflects a complex calculus performed over multiple independent features of an event. Thus, the actions of an agent like Hannibal Lecter may be perceived as simultaneously harmful and impure. The apparent categorical nature of moral domains may be an artifact of past research, including our own. This research has emphasized differences in moral judgment by comparing domain “exemplars” such as murder and incest, while paying less attention to features of behaviors that may blur domain boundaries. Future work should aim to examine moral attitudes in response to a larger and more diverse set of behaviors, including those that do not obviously fall into one domain or another.

Future research should also move beyond the investigation of harm and purity to a broader investigation of the cues that determine membership in other moral domains, including fairness, in-group loyalty, and respect for authority [2,3,23]. Here we examined the cues of action and target in determining domain membership with respect to harm and purity, but other cues may be more relevant for other domains. For example, perceiving fairness and authority violations may hinge on knowledge of the *relationship* between the interacting agents [56,57]. While relationship knowledge is undoubtedly important for judging the severity of harm violations, it may be less critical for determining whether or not harm occurred in the first place. In addition, judgments of in-group loyalty necessitate knowledge of the agent’s group membership but not necessarily the agent’s relationship to a specific target or victim (e.g., *washing one*’s *toilet with the US flag* [2,6]). The current work represents a first step in understanding not only how moral judgments differ across domains but also how these domains are determined in the first place.

## Supporting Information

Figure S1 **Perceived harmfulness and impurity, separate measures.** Separate judgments of harmfulness (anger, damage) and impurity (disgust, unnaturalness), in raw form (1.a.) as well as standardized, controlling for effects on the opposing measure (1.b.). Error bars represent ± 1 SE.
(TIF)

Text S1 **Additional Analyses.** In the main text, we report the effects of target and action on composite measures of perceived harmfulness (anger and damage judgments) and perceived impurity (disgust and unnaturalness judgments). Here we report results from two mixed effects ANOVAs on the individual measures used to construct the composites.
(DOCX)

Text S2 **Stimuli and Measures**. Stimuli and dependent measures used in experiments 1 and 2 are reported in full.
(DOCX)
