# Correction: Harming Ourselves and Defiling Others: What Determines a Moral Domain?

**DOI:** 10.1371/annotation/38818ce6-1b40-4965-aa64-b7943d2711ed

**Published:** 2013-09-30

**Authors:** Alek Chakroff, James Dungan, Liane Young

There was an error in the author contributions section of the article. A correct version of the section is available below.

Conceived and designed the experiments: AC JD LY. Performed the experiments: AC. Analyzed the data: AC. Wrote the manuscript: AC JD LY. 

