# Supplementary figures and images for: Harming Ourselves and Defiling Others: What Determines a Moral Domain?
Source: PLoS One. 2013 Sep 11;8(9):e74434. doi: 10.1371/journal.pone.0074434 (PMC3770666; doi:10.1371/journal.pone.0074434)

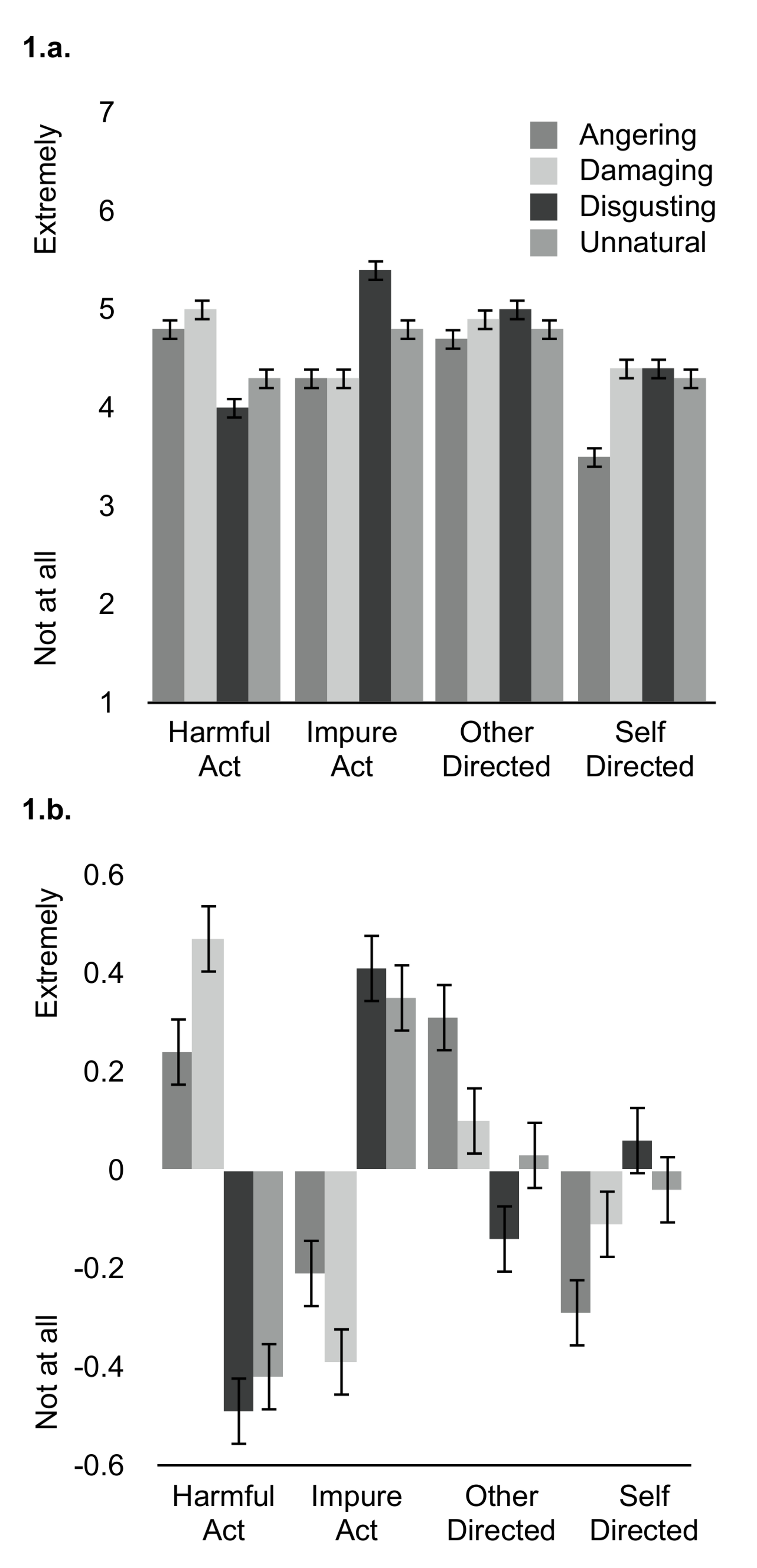

Supplement: Figure S1 — Perceived harmfulness and impurity, separate measures. Separate judgments of harmfulness (anger, damage) and impurity (disgust, unnaturalness), in raw form (1.a.) as well as standardized, controlling for effects on the opposing measure (1.b.). Error bars represent ± 1 SE. (TIF) [file pone.0074434.s001.tif]
