# Supplementary material for: Harming Ourselves and Defiling Others: What Determines a Moral Domain?
Source: PLoS One. 2013 Sep 11;8(9):e74434. doi: 10.1371/journal.pone.0074434 (PMC3770666; doi:10.1371/journal.pone.0074434)
Supplement: Text S1 — Additional Analyses. In the main text, we report the effects of target and action on composite measures of perceived harmfulness (anger and damage judgments) and perceived impurity (disgust and unnaturalness judgments). Here we report results from two mixed effects ANOVAs on the individual measures used to construct the composites. (DOCX) [file pone.0074434.s002.docx]

**Text S1. Additional Analyses**

In the main text, we report the effects of target and action on composite measures of perceived harmfulness (anger and damage judgments) and perceived impurity (disgust and unnaturalness judgments). Here we report results from two mixed effects ANOVAs on the individual measures used to construct the composites. We first report results from an analysis of affective measures: a 2 (action: harmful versus impure) x 2 (target: other-directed versus self-directed) x 2 (measure: anger versus disgust) ANOVA. We then report results from an analysis of action appraisals: a 2 (action: harmful versus impure) x 2 (target: other-directed versus self-directed) x 2 (measure: damage versus unnaturalness) ANOVA. Effects of action and target on each measure are shown in Figure S1.

In brief, the critical interactions involving action (harmful versus impure) and target (other-directed versus self-directed), as reported in the main text, remain significant when we use the emotional measures of anger and disgust. The results are mixed when we use the measures of damage and unnaturalness.

*Experiment 1, Prediction 1: Anger and Disgust*

*Main Effects*. Participants judged acts to be more disgusting (*M* = 4.7, *SE* = .09) than angering (*M* = 4.1, *SE* = .1) on the whole (main effect of measure; *F*(1, 327) = 86.22, *p* < .001, η_p_^2^ = .2). Participants also delivered “harsher” judgments (more disgusting and angering) for impure acts (*M* = 4.9, *SE* = .11) versus harmful acts (*M* = 3.9, *SE* = .12) (main effect of action; *F*(1, 327) = 32.08, *p* < .001, η_p_^2^ = .09). Finally, participants delivered “harsher” judgments (more disgusting and angering) for other-directed acts (*M* = 4.8, *SE* = .12) versus self-directed acts (*M* = 4.0, *SE* = .12) (main effect of target; *F*(1, 327) = 26.07, *p* < .001, η_p_^2^ = .07).

*Action, Anger and Disgust*. An interaction emerged between action and measure (*F*(1, 327) = 45.0, *p* < .001, η_p_^2^ = .12): impure acts were judged as more disgusting (*M* = 5.4, *SE* = .12) than angering (*M* = 4.3, *SE* = .13) (*t*(180) = 9.72, *p* < .001), and more disgusting as compared to harmful acts (*M* = 4.0, *SE* = .13) (*t*(329)=-8.22, *p* < .001). Harmful acts were also judged as more disgusting (*M* = 4.0, *SE* = .13) than angering (*M* = 3.8, *SE* = .14), but to a lesser extent (*t*(149) = 2.22, *p* = .03). Finally, impure acts were judged as more angering (*M* = 4.3, *SE* = .13) than harmful acts (*M* = 3.8, *SE* = .14) (*t*(329) = -2.42, *p* = .02).

*Target, Anger and Disgust*. An interaction emerged between target and measure (*F*(1, 327) = 22.84, *p* < .001, η_p_^2^ = .07): self-directed acts were judged as more disgusting (*M* = 4.4, *SE* = .12) than angering (*M* = 3.5, *SE* = .14) (*t*(161) = 8.0, *p* < .001), but as less disgusting as compared to other-directed acts (*M* = 5.0, *SE* = .11) (*t*(329) = -2.76, *p* = .006). Other-directed acts were also judged as more disgusting (*M* = 5.0, *SE* = .11) than angering (*M* = 4.7, *SE* = .14) but to a lesser extent (*t*(168) = 4.72, *p* < .001), as reflected in the significant interaction. Other-directed acts were also judged as more angering as compared to self-directed acts (*M* = 3.5, *SE* = .14) (*t*(329) = -6.2*, p* < .001). Notably, the effect of target was stronger on the anger versus disgust measure, also reflected in the significant target x measure interaction.

Some of these effects are difficult to interpret, due to unpredicted main effects on our measures. We therefore note that, because the disgust and anger measures are highly related (*r*(331) = .83, *p* < .001), it is also useful to examine the unique effects of our manipulations on each measure, controlling for the effects on the other measure (see Gutierrez & Giner-Sorolla, 2007; Russell & Giner-Sorolla, 2011c; Giner-Sorolla et al., 2012). We thus conducted the same ANOVA presented above on measures capturing unique variance in disgust and anger. We computed these measures as the standardized residual values in disgust, predicted from anger, and vice versa.

As above, an interaction emerged between action and measure (*F*(1, 327) = 53.77, *p* < .001, η_p_^2^ = .14). Harmful acts were judged as more angering (*M* = .24, *SE* = .08) than disgusting (*M* = -.49, *SE* = .07) (*t*(149) = 6.87, *p* < .001), and more angering as compared to impure acts (*M* = -.21, *SE* = .07) (*t*(329) = 4.1, *p* < .001). By contrast, impure acts were judged as more disgusting (*M* = .41, *SE* = .07) than angering (*M* = -.21, *SE* = .07) (*t*(180) = -4.1, *p* < .001), and more disgusting as compared to harmful acts (*M* = -.49, *SE* = .07) (*t*(329) = -9.1, p < .001).

Also, as above, an interaction emerged between target and measure (*F*(1, 327) = 18.9, *p* < .001, η_p_^2^ = .06). Other-directed acts were judged as more angering (*M* = .31, *SE* = .07) than disgusting (*M* = -.14, *SE* = .07) (*t*(168) = 3.98, p < .001), and as more angering as compared to self-directed acts (*M* = -.29, *SE* = .07) (*t*(329) = -5.76, *p* < .001). By contrast, self-directed acts were judged as more disgusting (*M* = .06, *SE* = .07) than angering (*M* = -.29, *SE* = .07) (*t*(161) = -2.4, *p* = .018), and as marginally more disgusting as compared to other-directed acts (*M* = -.14, *SE* = .07) (*t*(329) = 1.92, *p* = .056). This is the same critical pattern of results observed in analyses on composite measures of harmfulness and impurity reported in the main text.

Finally, there were no significant interactions between action and target (*F*(1, 327) = .70, *p* = .41, η_p_^2^ = .002), or between action, target and measure (*F*(1, 327) = 1.58, *p* = .21, η_p_^2^ = .005).

*Experiment 1, Prediction 1: Damage and Unnaturalness*

*Main Effects*. Participants judged acts to be similarly unnatural (*M* = 4.6, *SE* = .09) and damaging (*M* = 4.7, *SE* = .08) (main effect of measure; *F*(1, 327) = 3.21, *p* = .07, η_p_^2^ = .01), and similarly “harsh” judgments (both unnatural and damaging) for impure acts (*M* = 4.5, *SE* = .11) and harmful acts (*M* = 4.7, *SE* = .12) (main effect of action; *F*(1, 327) = .54, *p* = .46, η_p_^2^ = .002). Finally, participants delivered “harsher” judgments (both unnatural and damaging) for other-directed acts (*M* = 4.8, *SE* = .11) versus self-directed acts (*M* = 4.4, *SE* = .11) (main effect of target; *F*(1, 327) = 9.19, *p* = .003, η_p_^2^ = .03).

*Action, Damage and Unnaturalness*. An interaction emerged between action and measure (*F*(1, 327) = 79.37, *p* < .001, η_p_^2^ = .2): harmful acts were judged as more damaging (*M* = 5.0, *SE* = .12) than unnatural (*M* = 4.3, *SE* = .14) (*t*(149) = -6.47, *p* < .001), and also more damaging as compared to impure acts (*M* = 4.3, *SE* = .11) (*t*(329) = 8.22, *p* < .001). In contrast, impure acts were judged as more unnatural (*M* = 4.8, *SE* = .12) than damaging (*M* = 4.3, *SE* = .11) (*t*(180) = 5.93, *p* < .001), and also more unnatural as compared to harmful acts (*M* = 4.3, *SE* = .14) (*t*(329) = 2.58, *p* = .01).

*Target, Damage and Unnaturalness*. There was no significant interaction between target and measure (*F*(1, 327) = .22, *p* = .64 , η_p_^2^ = .001): self-directed acts were judged as similarly unnatural (*M* = 4.3, *SE* = .14) and damaging (*M* = 4.4, *SE* = .13) (*t*(161) = -.29, *p* = .78). Other-directed acts were also judged as similarly unnatural (*M* = 4.8, *SE* = .12) and damaging (*M* = 4.9, *SE* = .11) (*t*(168) = -.99, *p* = .32). Finally, the effect of target was similarly strong for judgments of unnaturalness (*t*(329) = -2.52, *p* = .01) and damage (*t*(329) = -3.15, *p* = .002).

Finally, there were no significant interactions between action and target (*F*(1, 327) = .54, *p* = .46, η_p_^2^ = .002), or between action, target and measure (*F*(1, 327) = .77, *p* = .38, η_p_^2^ = .002).

We again examined the unique effects of our manipulations on each measure, controlling for the effects on the other measure. As above, we computed these measures as the standardized residual values in unnaturalness, predicted from damage, and vice versa.

As above, an interaction emerged between action and measure (*F*(1, 327) = 82.63, *p* < .001, η_p_^2^ = .2). Harmful acts were judged as more damaging (*M* = .47, *SE* = .07) than unnatural (*M* = -.42, *SE* = .09) (*t*(149) = -6.09, *p* < .001), and more damaging as compared to impure acts (*M* = -.39, *SE* = .07) (*t*(329) = 8.58, *p* < .001). In contrast, impure acts were judged as more unnatural (*M* = .35, *SE* = .06) than damaging (*M* = -.39, *SE* = .07) (*t*(180) = ,6.72 *p* < .001), and as more unnatural as compared to harmful acts (*M* = -.42, *SE* = .09) (*t*(329) = -7.56, *p* < .001).

As above, there was no interaction between target and measure (*F*(1, 327) = .61, *p* .44, η_p_^2^ = .002). Other-directed acts were judged as similarly damaging (*M* = .10, *SE* = .07) and unnatural (*M* = .03, *SE* = .07) (*t*(168) = -.54, p = .59), but as more damaging as compared to self-directed acts (*M* = -.11, *SE* = .08) (*t*(329) = -1.96, *p* = .05). Self-directed acts were also judged as similarly damaging (*M* = -.11, *SE* = .08) and unnatural (*M* = -.04, *SE* = .08) (*t*(161) = ,.49 *p* = .63), and as similarly unnatural as compared to other-directed acts (*M* = .03, *SE* = .07) (*t*(329) = -.63, *p* = .53).

Finally, as above, there were no significant interactions between action and target (*F*(1, 327) = .57, *p* = .45, η_p_^2^ = .002), or between action, target and measure (*F*(1, 327) = .93, *p* = .34, η_p_^2^ = .003).

**Stimuli and Measures**

**Study 1: Stimuli**

*Participants were randomly assigned to one of the following eight conditions. Participants read each of the four items on separate pages, with order randomized.*

Other-directed Intentional Harmful Act

- Imagine that Steven intentionally punched someone in the ribs.

- Imagine that Steven intentionally cut someone on the arm with a sharp knife.

- Imagine that Steven intentionally closed a door on someone's fingers.

- Imagine that Steven intentionally poured a cup of painfully hot water on someone's lap.

Other-directed Intentional Impure Act

- Imagine that Steven intentionally poured a cup of urine on someone's lap.

- Imagine that Steven intentionally smeared cat poop on someone's arm.

- Imagine that Steven intentionally bought dog meat at a foreign grocery store and served it to someone.

- Imagine that Steven intentionally ordered stir-fried rat for someone at a small restaurant in the city.

Other-directed Accidental Harmful Act

- Imagine that Steven accidentally punched someone in the ribs.

- Imagine that Steven accidentally cut someone on the arm with a sharp knife.

- Imagine that Steven accidentally closed a door on someone's fingers.

- Imagine that Steven accidentally poured a cup of painfully hot water on someone's lap.

Other-directed Accidental Impure Act

- Imagine that Steven accidentally poured a cup of urine on someone's lap.

- Imagine that Steven accidentally smeared cat poop on someone's arm.

- Imagine that Steven accidentally bought dog meat at a foreign grocery store and served it to someone.

- Imagine that Steven accidentally ordered stir-fried rat for someone at a small restaurant.

Self-directed Intentional Harmful Act

- Imagine that Steven intentionally punched himself in the ribs.

- Imagine that Steven intentionally cut himself on the arm with a sharp knife.

- Imagine that Steven intentionally closed a door on his own fingers.

- Imagine that Steven intentionally poured a cup of painfully hot water on his own lap.

Self-directed Intentional Impure Act

- Imagine that Steven intentionally poured a cup of urine on his own lap.

- Imagine that Steven intentionally smeared cat poop on his own arm.

- Imagine that Steven intentionally bought dog meat at a foreign grocery store and ate it.

- Imagine that Steven intentionally ordered stir-fried rat for himself at a small restaurant.

Self-directed Accidental Harmful Act

- Imagine that Steven accidentally punched himself in the ribs.

- Imagine that Steven accidentally cut himself on the arm with a sharp knife.

- Imagine that Steven accidentally closed a door on his own fingers.

- Imagine that Steven accidentally poured a cup of painfully hot water on his own lap.

Self-directed Accidental Impure Act

- Imagine that Steven accidentally poured a cup of urine on his own lap.

- Imagine that Steven accidentally smeared cat poop on his own arm.

- Imagine that Steven accidentally bought dog meat at a foreign grocery store and ate it.

- Imagine that Steven accidentally ordered stir-fried rat for himself at a small restaurant.

**Experiment 1: Measures**

*Participants made the following ratings on a single page below each scenario. Moral judgments [either 1.a or 1.b] were presented first; order of other judgments was randomized across trials. All judgments were made on 7-pt likert scales.*

1.a How morally wrong is this behavior?

[Not at all wrong 1 - 7 Extremely wrong]

1.b How immoral is Steven as a person?

[Not at all immoral 1 - 7 Extremely immoral]

1. How much does this violate the natural order of things - how unnatural is it?

[Not at all unnatural 1 - 7 Extremely unnatural]

1. How disgusted do you feel about this?

[Not at all disgusted 1 - 7 Extremely disgusted]

1. How damaging is this?

[Not at all damaging 1 - 7 Extremely damaging]

1. How angry do you feel about this?

[Not at all angry 1 - 7 Extremely angry]

**Experiment 2: Stimuli**

*Participants were assigned to one of four conditions, reading two scenarios, order counterbalanced across participants.*

Self-directed Harmful Act

- John once cut himself with a knife when he was upset.

- Robert once closed a door on his own fingers when he was upset.

Other-directed Harmful Act

- John once cut someone with a knife when he was upset.

- Robert once closed a door on someone's fingers when he was upset.

Self-directed Impure Act

- John once found a dead dog on the road and cooked it up for dinner.

- Robert once smeared cat poop on his face for fun.

Other-directed Impure Act

- John once found a dead dog on the road, cooked it up and fed it to someone else for dinner.

- Robert once smeared cat poop on someone's face for fun.

**Experiment 2: Measures**

*Participants made three moral judgments focusing on the action, and three moral judgments focusing on the character. Moral judgments were made in blocks, block order was counterbalanced across participants. All judgments were made on 100-point slider scales.*

Action Focus

- Were these actions morally blameworthy?

- Were these actions deserving of punishment?

- Were these actions immoral?

[Not at all 0 --------100 Absolutely]

Character Focus

- Is John "screwed up"?

- Is john "sick and twisted"?

- Is John likely to have normal human feelings?

[Not at all 0 --------100 Absolutely]
