# Supplementary material for: Harming Ourselves and Defiling Others: What Determines a Moral Domain?
Source: PLoS One. 2013 Sep 11;8(9):e74434. doi: 10.1371/journal.pone.0074434 (PMC3770666; doi:10.1371/journal.pone.0074434)
Supplement: Text S2 — Stimuli and Measures. Stimuli and dependent measures used in experiments 1 and 2 are reported in full. (DOCX) [file pone.0074434.s003.docx]

**Text S2. Stimuli and Measures**

**Study 1: Stimuli**

*Participants were randomly assigned to one of the following eight conditions. Participants read each of the four items on separate pages, with order randomized.*

Other-directed Intentional Harmful Act

- Imagine that Steven intentionally punched someone in the ribs.

- Imagine that Steven intentionally cut someone on the arm with a sharp knife.

- Imagine that Steven intentionally closed a door on someone's fingers.

- Imagine that Steven intentionally poured a cup of painfully hot water on someone's lap.

Other-directed Intentional Impure Act

- Imagine that Steven intentionally poured a cup of urine on someone's lap.

- Imagine that Steven intentionally smeared cat poop on someone's arm.

- Imagine that Steven intentionally bought dog meat at a foreign grocery store and served it to someone.

- Imagine that Steven intentionally ordered stir-fried rat for someone at a small restaurant in the city.

Other-directed Accidental Harmful Act

- Imagine that Steven accidentally punched someone in the ribs.

- Imagine that Steven accidentally cut someone on the arm with a sharp knife.

- Imagine that Steven accidentally closed a door on someone's fingers.

- Imagine that Steven accidentally poured a cup of painfully hot water on someone's lap.

Other-directed Accidental Impure Act

- Imagine that Steven accidentally poured a cup of urine on someone's lap.

- Imagine that Steven accidentally smeared cat poop on someone's arm.

- Imagine that Steven accidentally bought dog meat at a foreign grocery store and served it to someone.

- Imagine that Steven accidentally ordered stir-fried rat for someone at a small restaurant.

Self-directed Intentional Harmful Act

- Imagine that Steven intentionally punched himself in the ribs.

- Imagine that Steven intentionally cut himself on the arm with a sharp knife.

- Imagine that Steven intentionally closed a door on his own fingers.

- Imagine that Steven intentionally poured a cup of painfully hot water on his own lap.

Self-directed Intentional Impure Act

- Imagine that Steven intentionally poured a cup of urine on his own lap.

- Imagine that Steven intentionally smeared cat poop on his own arm.

- Imagine that Steven intentionally bought dog meat at a foreign grocery store and ate it.

- Imagine that Steven intentionally ordered stir-fried rat for himself at a small restaurant.

Self-directed Accidental Harmful Act

- Imagine that Steven accidentally punched himself in the ribs.

- Imagine that Steven accidentally cut himself on the arm with a sharp knife.

- Imagine that Steven accidentally closed a door on his own fingers.

- Imagine that Steven accidentally poured a cup of painfully hot water on his own lap.

Self-directed Accidental Impure Act

- Imagine that Steven accidentally poured a cup of urine on his own lap.

- Imagine that Steven accidentally smeared cat poop on his own arm.

- Imagine that Steven accidentally bought dog meat at a foreign grocery store and ate it.

- Imagine that Steven accidentally ordered stir-fried rat for himself at a small restaurant.

**Experiment 1: Measures**

*Participants made the following ratings on a single page below each scenario. Moral judgments [either 1.a or 1.b] were presented first; order of other judgments was randomized across trials. All judgments were made on 7-pt likert scales.*

1.a How morally wrong is this behavior?

[Not at all wrong 1 - 7 Extremely wrong]

1.b How immoral is Steven as a person?

[Not at all immoral 1 - 7 Extremely immoral]

1. How much does this violate the natural order of things - how unnatural is it?

[Not at all unnatural 1 - 7 Extremely unnatural]

1. How disgusted do you feel about this?

[Not at all disgusted 1 - 7 Extremely disgusted]

1. How damaging is this?

[Not at all damaging 1 - 7 Extremely damaging]

1. How angry do you feel about this?

[Not at all angry 1 - 7 Extremely angry]

**Experiment 2: Stimuli**

*Participants were assigned to one of four conditions, reading two scenarios, order counterbalanced across participants.*

Self-directed Harmful Act

- John once cut himself with a knife when he was upset.

- Robert once closed a door on his own fingers when he was upset.

Other-directed Harmful Act

- John once cut someone with a knife when he was upset.

- Robert once closed a door on someone's fingers when he was upset.

Self-directed Impure Act

- John once found a dead dog on the road and cooked it up for dinner.

- Robert once smeared cat poop on his face for fun.

Other-directed Impure Act

- John once found a dead dog on the road, cooked it up and fed it to someone else for dinner.

- Robert once smeared cat poop on someone's face for fun.

**Experiment 2: Measures**

*Participants made three moral judgments focusing on the action, and three moral judgments focusing on the character. Moral judgments were made in blocks, block order was counterbalanced across participants. All judgments were made on 100-point slider scales.*

Action Focus

- Were these actions morally blameworthy?

- Were these actions deserving of punishment?

- Were these actions immoral?

[Not at all 0 --------100 Absolutely]

Character Focus

- Is John "screwed up"?

- Is john "sick and twisted"?

- Is John likely to have normal human feelings?

[Not at all 0 --------100 Absolutely]
